# Assessment of Species Diversity and Distribution of an Ancient Diatom Lineage Using a DNA Metabarcoding Approach

**DOI:** 10.1371/journal.pone.0103810

**Published:** 2014-08-18

**Authors:** Deepak Nanjappa, Stephane Audic, Sarah Romac, Wiebe H. C. F. Kooistra, Adriana Zingone

**Affiliations:** 1 Stazione Zoologica Anton Dohrn, Naples, Italy; 2 CNRS, UMR EPEP – Évolution des Protistes et des Écosystèmes Pélagiques, UPMC Sorbonne Universités, Station Biologique de Roscoff, Roscoff, France; American University in Cairo, Egypt

## Abstract

**Background:**

Continuous efforts to estimate actual diversity and to trace the species distribution and ranges in the natural environments have gone in equal pace with advancements of the technologies in the study of microbial species diversity from microscopic observations to DNA-based barcoding. DNA metabarcoding based on Next Generation Sequencing (NGS) constitutes the latest advancement in these efforts. Here we use NGS data from different sites to investigate the geographic range of six species of the diatom family Leptocylindraceae and to identify possible new taxa within the family.

**Methodology/Principal Findings:**

We analysed the V4 and V9 regions of the nuclear-encoded SSU rDNA gene region in the NGS database of the European ERA-Biodiversa project BioMarKs, collected in plankton and sediments at six coastal sites in European coastal waters, as well as environmental sequences from the NCBI database. All species known in the family Leptocylindraceae were detected in both datasets, but the much larger Illumina V9 dataset showed a higher species coverage at the various sites than the 454 V4 dataset. Sequences identical or similar to the references of *Leptocylindrus aporus*, *L. convexus*, *L. danicus/hargravesii* and *Tenuicylindrus belgicus* were found in the Mediterranean Sea, North Atlantic Ocean and Black Sea as well as at locations outside Europe. Instead, sequences identical or close to that of *L. minimus* were found in the North Atlantic Ocean and the Black Sea but not in the Mediterranean Sea, while sequences belonging to a yet undescribed taxon were encountered only in Oslo Fjord and Baffin Bay.

**Conclusions/Significance:**

Identification of Leptocylindraceae species in NGS datasets has expanded our knowledge of the species biogeographic distribution and of the overall diversity of this diatom family. Individual species appear to be widespread, but not all of them are found everywhere. Despite the sequencing depth allowed by NGS and the wide geographic area covered by this study, the diversity of this ancient diatom family appears to be low, at least at the level of the marker used in this study.

## Introduction

Diversity assessments of eukaryotic unicellular organisms, commonly referred to as protists, are often constrained by their restricted number of morphological features that can be used for species differentiation, as well as by morphological stasis and phenotypic plasticity, which affect proper taxonomic assignment. Molecular approaches have aided a delimitation of taxa based on more objective data and an ad hoc assessment of the morphology that fits the molecular data. The results of such studies have shown that protistan biodiversity is far higher than what can be appreciated with morphological means alone. While some cases of phenotypic plasticity have been identified (e.g. [1]), the majority of molecular-based taxonomic revisions points at cryptic or pseudo-cryptic species, particularly in diatoms, implying that the actual diversity of these microorganisms is underestimated [2]. The utility of describing cryptic and pseudo-cryptic species and tracing their geographic and ecological ranges is demonstrated by the observations that such species can have different biochemical [3] and physiological characteristics [4], [5], which may underpin different seasonal and spatial ranges [6], [7].

Discovery of new microalgal species has generally relied on isolation and cultivation of unialgal strains. However, sheer logistics limit the number of strains that can be scrutinized, while rare, minute, or unculturable species escape identification. At the same time, ecological and biogeographic studies suffer from the limitation of tracing cryptic or difficult-to-identify species in natural samples. The way forward towards improved insight in protistan biodiversity and distribution is to integrate classical methods with molecular detection approaches. Several such methods are available. Amongst these are FISH, qPCR, and microarray screening, which, however, are able to track only known taxa. Metabarcoding of PCR-amplified discriminative nucleotide markers from environmental DNA allows tracing these organisms directly in their environment without the need for cultivation. In this way spatial patterns and seasonal distribution can be reconstructed and diversity explored more extensively by using sequences as proxies. The latter approach also enables discovery of new taxa associated to newfound sequences that cannot be assigned to any known organism. DNA metabarcoding was initially performed through Sanger-sequencing of clone libraries [8]–[10]. More recently NGS methods have greatly increased the number of sequences obtained from individual samples, thus overcoming the bias against rare taxa. The method, initially applied to bacteria (e.g. [11], [12]) has been extended to protists [13], [14] and pursued worldwide [15], [16], giving unprecedented opportunities to explore diversity and geographic ranges of microbial organisms.

The use of NGS on PCR-amplified environmental DNA has so far addressed the total diversity and spatial patterns of whole groups of planktonic protists (e.g. [17], [18]). In the present NGS study we explore the biodiversity of a planktonic diatom family, the Leptocylindraceae, and the distribution of its member species. These species are common from polar to sub-tropical coastal regions, where they often are prominent constituents of diatom blooms. Combined morphological and genetic studies of cultured strains from the Gulf of Naples (GoN) by Nanjappa and co-workers [7] delineated five species within *Leptocylindrus*, two of which, *L. danicus* and *L. minimus*, were already known [19], and three, *L. hargravesii*, *L. aporus* and *L. convexus*, were new to science. An additional species was assigned to the genus *Tenuicylindrus* as *T. belgicus* [20]. The four new species only differed from those already known by subtle morphological details and were probably misidentified in previous studies [7]. Molecular phylogenies resolved *Leptocylindrus* and *Tenuicylindrus* as sisters at a basal position in the radial centrics, the group of diatoms with the most ancient fossil record, suggesting that these genera constitute a poorly diverse remnant of a once far more diverse lineage. Alternatively, the paucity of species may be the result of a single locality investigated, the GoN, and only with a cultivation approach.

The distribution and the local diversity of the species so far identified in the Leptocylindraceae cannot be reconstructed from previous records, which were mostly based on light microscopy identification of two previously ill-defined taxa, *L. danicus* and *L. minimus*. Here we use DNA metabarcoding, NGS data from several sites across European seas collected in the frame of the ERA Biodiversa project BioMarKs (http://www.biomarks.eu) as well as sequences deposited in the public nucleotide sequence database GenBank, with the aim of assessing the diversity of leptocylindracean species and exploring their biogeographic range. As marker we chose the nuclear-encoded SSU rDNA because of the availability of an ever-growing dataset of taxonomically validated reference sequences, covering virtually every known eukaryotic lineage [21]. As sequence length is still a restricting factor in NGS approaches, we sequenced two short variable regions in the SSU rDNA, namely the V4 and V9 tags, for which universal amplification primers are available. Leptocylindraceae are good candidates for this attempt to use NGS data to track diatom diversity in environmental samples because the SSU rDNA sequence differs notably among the known species in the family, with the only exception of *L. danicus* and *L. hargravesii*, which only differ at a few positions. On the other hand the considerable distances among sequences of the known species and their basal position in the phylogeny of diatoms [7] pose some problems in the selection of the adequate cut-off for the retrieval of sequences belonging to the family.

## Methods

### Ethics statement

No specific permissions were required to collect surface phytoplankton samples at six coastal stations across Europe (GPS coordinates are provided in Table S1 in Tables S1). The study did not involve endangered or protected species.

### Definition of the query datasets

Two types of SSU rDNA datasets were used in this study. The first consisted of sequences of the V4 region (390 bp), obtained through 454 pyrosequencing, and of the V9 region (130 bp), obtained through Illumina sequencing, gathered from planktonic and benthic samples collected within the ERA Biodiversa project BioMarKs (http://www.biomarks.eu/). The second comprised the nucleotide sequences deposited in GenBank (http://www.ncbi.nlm.nih.gov/genbank/) which, in addition to sequences from cultivated strains, also includes environmental sequences of known geographic provenance.

Regarding the BioMarKs dataset, we examined sequences obtained from coastal stations at six localities along the European coasts. These included Naples (Long Term Ecological Research station MareChiara, LTER-MC, Tyrrhenian Sea) and Blanes Bay (Blanes Bay Microbial Observatory, BBMO) in the Mediterranean Sea; Oslo Fjord, Roscoff (station SOMLIT-Astan, Western English Channel) and Gijon (Spain), on the North-eastern Atlantic coast; and Varna on the Black Sea (see Table S1 in Tables S1 for metadata). Plankton and sediments samples were collected at all these stations during 2010. Additional plankton and sediment samples from the stations in Naples and Oslo Fjord were obtained during the fall of 2009. BioMarKs protocols for sampling, sequencing and processing of reads have been described in Logares et al. [22]. Briefly, seawater samples were taken with Niskin bottles from near the surface (subsurface, 1 m) and at depth (20–40 m). Irrespective of the presence of a chlorophyll maximum, the latter samples were indicated as DCM (Deep Chlorophyll Maximum) in the dataset. Plankton samples were size-fractioned into 0.8–3 µm, 3–20 µm and 20–2000 µm samples on polycarbonate filters (142 mm and 47 mm diameter). Immediately upon filtration, filters were flash-frozen in liquid N_2_ and stored at −80°C. Sediment samples were gathered using sediment corers and small aliquots from the surface layer were frozen at −80°C. Total DNA and RNA in plankton samples were extracted from the same filter using the NucleoSpin RNA kit (Macherey-Nagel, Hoerdt, France). Total DNA and RNA were extracted from sediment samples using the RNA Power Soil Total Isolation kit combined with DNA Elution Accessory kit (MoBio Laboratories). Extracted RNA was reverse transcribed to cDNA using the RT Superscript III random primers kit (Invitrogen, Carlsbad, CA, USA).

The V4 sequence dataset was generated from each of the three size fractions of each plankton sample (at two water depths) and from sediment samples for all sites and collection dates, with sequences originating from the DNA as well as from the cDNA. The V4 region was PCR-amplified using eukaryote-specific primers [13] and sequenced at the CEA Genoscope in Evry (France) using a GS FLX emPCR Genomic Lib-L kit according to the manufacturer's protocol (Genome Sequencer FLX Titanium, 454 Life Sciences from Roche, Brandford, CT, USA). For protocols of V4 PCR, sequencing, quality filtering and curation of sequences refer to Logares *et al.* [22].This was the first dataset produced in BioMarKs, and some samples, namely those from Oslo Fjord 2009 and Naples 2009, were sequenced multiple times.

The V9 dataset was generated from three size fractions of surface samples and largely cDNA as template, and both DNA and cDNA templates of sediment samples (Table S4 in Tables S1). The V9 region was PCR-amplified using the universal forward primer 1389F and the eukaryotic-specific reverse primer 1510R [23]. Amplifications of the V9 region were done in triplicate using the following PCR program: initial denaturation step at 98°C for 30 sec, followed by 25 cycles of 10 sec at 98°C, 30 sec at 57°C, 30 sec at 72°C, followed by 15 cycles of 10 sec at 98°C, 30 sec at 48°C, 30 sec at 72°C and final elongation step at 72°C for 10 minutes. Amplicons were then pooled and purified using the NucleoSpin® Extract II kit (Macherey-Nagel, Hoerdt, France). To obtain a similar number of reads for each sample, purified amplicons were quantified with the Quant-iT PicoGreen dsDNA kit (Invitrogen) and then mixed in equal concentrations. Bridge amplification and sequencing were performed using a Genome Analyser IIx system (Illumina, San Diego, CA, USA) at Genoscope - Centre National de Séquençage (Evry, France). Overlapping sequencing reads were merged using an internal script based on the fastx library (http://hannonlab.cshl.edu/fastx_toolkit/index.html). Forward and reverse primer sequences were detected in the reads and the region between them was extracted. Extracted sequences were quality filtered and chimeras were detected using the chimera search module of the usearch program [24], looking for chimeras with respect to a reference database, and also within each sample. This was the second dataset produced in the BioMarKs project.

### Gathering leptocylindracean sequences from the datasets

Bacillariophycean sequences were retrieved from the BioMarKs V4 and V9 datasets according to the following procedure. We used two reference datasets derived from the PR2 database [21] and truncated to the boundary of the targeted amplicon (V4 or V9), excluding the primer sequence. Environmental sequences were searched against the corresponding reference dataset using a global alignment program (ggsearch36) (http://faculty.virginia.edu/wrpearson/fasta/CURRENT/) from the fasta package. For each query sequence, we retained in this initial assignation the assignation of the best hit (based on percentage of identity of the global alignment), or the assignation of the last common ancestor of the best hits in case of equality. Environmental sequences with assignation matching Bacillariophyta formed our initial dataset.

The BioMarKs V4 bacillariophycean sequences were clustered with V4 reference sequences of two bolidophyceans used as outgroups and 102 diatoms, including Leptocylindraceae, as in [7], with the CD-HIT-EST-2D module of CD-HIT suite (http://weizhong-lab.ucsd.edu/cdhit_suite/cgi-bin/index.cgi). Likewise, the bacillariophycean BioMarKs V9 sequences were clustered with V9 reference-sequences of two bolidophyceans and 95 diatoms. The settings of the clustering procedure were as follows: sequence identity cut-off value set at 0.90 (see Methods S1 for rationale of choice); do not compare strands; use global sequence identity; cluster sequence to the best cluster that meets the threshold; use bandwidth of 20. Alignment coverage parameters and length coverage parameters were set to defaults.

All sequences in the query dataset with similarity ≥0.90 to any of the references were matched with the most closely related reference with CD-HIT-EST-2D. Clusters of sequences grouping closer to non-leptocylindracean references were eliminated, whereas clusters that grouped closer to any one of the leptocylindracean references were re-clustered at the ≥0.97 similarity level (other parameters, as before). The secondary clusters of sequences with similarity ≥0.97 are hereafter referred to as Operational Taxonomic Units (OTUs). OTUs represented by a single sequence (singleton) or two sequences (doubleton) were removed from the V4 and V9 datasets because these sequences have a high probability of representing sequence errors.

Putative leptocylindracean sequences were retrieved from the nucleotide collection in GenBank using the megaBLAST option (http://blast.ncbi.nlm.nih.gov/ accessed 16 August 2013). As queries we used the entire SSU rDNA reference sequence of the known leptocylindracean species [7]. Returned sequences were retained if they showed a similarity value to the query higher than, or equal to, the similarity of the most dissimilar leptocylindracean reference sequence to the query in the retrieved data. Returned sequences that differed markedly from the query sequences, but that were recovered within the leptocylindracean clade in a distance tree inferred from the returned sequences (distance tree option in BLAST), were used as queries in their own turn. Returned sequences with lower similarity values to the query than to the most dissimilar leptocylindracean sequence that was returned were considered to belong to species in other diatom families and were excluded. In cases of doubt, a returned sequence was used as query, and was discarded if its closest return sequences belonged to taxa other than Leptocylindraceae. The procedure was repeated with only the V4 region and only the V9 region to ensure inclusion of short SSU rDNA fragments similar to the queries.

### Taxonomic validation

To confirm that the obtained OTUs belonged to Leptocylindraceae, the sequences gathered as described above were aligned and analysed phylogenetically. One dataset included the V4 reference-sequences of leptocylindracean species and of other diatoms and of bolidomonads, as well as single representatives of putative leptocylindracean BioMarKs OTUs and all putative leptocylindracean sequences from GenBank. Only one representative sequence per BioMarKs OTU was included to keep the size of the dataset manageable. A V9 dataset was generated similarly. A third dataset was prepared with the full-length nuclear SSU rDNA sequences of all diatom and bolidomonad reference sequences used in [7] and putative leptocylindracean sequences retrieved from GenBank. Sequence alignment of these three datasets was done on the T-REX web server [25] using the slow and iterative refinement method (FFT-NS-i) [26] of the Multiple Sequences Alignment (MSA) programme MAFFT v6.864. Resulting alignments were curated in BioEdit v.7.2.0. [27].

The phylogenetic signal in the alignment of leptocylindracean sequences was assessed by comparing the skewedness of the tree-length distribution of 100,000 random trees calculated under parsimony settings in PAUP (Phylogenetic Analyses Using Parsimony; version 4.0 and other methods) [28] with threshold values for 4-state data in [29], given the number of parsimony informative sites and OTUs ( = distinct sequences) in the alignment.

A maximum likelihood (ML) approach was used to infer the phylogenetic position of each of the OTUs and GenBank sequences, and to determine which of these resolved within – or as nearest sister to – Leptocylindraceae. Trees were inferred from the three aligned datasets using RAxML-VI-HPC [30] on the T-REX web server [25]. The following settings were applied: substitution model, GTR-GAMMA; algorithm executed, Hill Climbing; 100 alternative bootstrap runs on distinct starting trees; bootstrap random seed-option (other options not modified). Trees were rooted with sequences of bolidomonads to identify the smallest-possible clade that included all of the leptocylindracean reference sequences. Resulting trees were visualized and edited in Dendroscope [31].

Sequences or OTUs included in the clade of all of the Leptocylindraceae reference- sequences were assigned to a named species when they resolved in the same clade as the reference sequence of that species. In case they resolved outside a terminal clade containing named reference sequences, sequences were queried in blastn and considered as false-positive when the nearest among the returned sequences belonged to species outside Leptocylindraceae. Otherwise they were treated as belonging to Leptocylindraceae. To assess the accuracy of the position of the OTUs without taxonomic reference sequences in the V4 and V9 trees, we compared those trees with a tree inferred from the whole SSU rDNA diatom reference sequence, assuming that the latter tree is closer to the actual diatom phylogeny.

### Geographic analysis

In order to normalise the number of sequences for a species amongst different sequence runs, the number of sequences assigned to that species was divided by the total number of sequences from that sample. This was repeated for each of the leptocylindracean species, for each of the size fractions in each of the plankton samples, for each of the sediment samples, for both DNA and cDNA, and for the V4 and the V9 sequences. To facilitate comparison of the abundance across different samples, the normalised abundances (% of the total sequences in the respective sequence run) of a group of samples of interest were summed and the sum normalized to 1 (100%).

### Statistical analyses of the dataset

To compare the total diversity across the stations, an OTU-based analysis was performed irrespective of the phylogenetic results using the whole dataset. A consensus taxonomic identity for the 454-V4 and Illumina-V9 sequences was obtained with ggsearch at a consensus confidence threshold of 80 [32]. Sequences belonging to Leptocylindraceae were gathered into a V4 and a V9 dataset. The two datasets were aligned with MSA program, MAFFT v6.864, using FFT-NS-i on the Trex web server and the obtained alignments were checked manually in BioEdit v.7.2.0. Pairwise similarities among the aligned sequences were calculated using Mothur (http://ww.mothur.org/). Based on the similarity matrix, sequences were clustered into OTUs at different clustering similarities, applying the rule of furthest neighbor and the highest precision (P = 1000). Rarefaction curves to relate numbers of harvested sequences with number of retrieved OTUs were obtained using Mothur. After removing OTUs that included only one or two sequences, with the remove.rare function, Venn diagrams were constructed to assess the diversity across the sampling sites using Mothur.

The abundance of sequences assignable to leptocylindraceans was compared between datasets of different sizes gathered from the same environmental sample, i.e., V9 versus V4 and cDNA versus DNA datasets, by calculating the expected number of sequences of one species in a sample downsampling each sequence samples to the size of the corresponding sample in the other dataset. The expected abundance, expressed as a range, was computed at a 0.05 significance level with the statistics proposed by Audic & Claverie [33].

## Results

### The datasets

An overview of sequence numbers for the BioMarKs datasets is presented in Table 1. The complete BioMarks Illumina V9 dataset (including both DNA and cDNA sequences from all fractions, sites and depths, also including metazoans) contained ca. 130 times more sequences than the 454 V4 dataset, and ca. 70 times more diatom sequences. Rarefaction curves constructed with the total Leptocylindraceae dataset (Figure S1 A & B) showed V4 OTUs reaching a plateau at a 97% similarity while V9 reached a plateau at 95%. Sequences initially assigned to Leptocylindraceae in the V9 dataset (using a 90% similarity threshold) were 30 times more abundant than in the V4 dataset. However, more than 2/3 of those sequences were removed because they either were more similar to other genera or could not be clearly assigned to Leptocylindraceae (false positives). Instead all of the 50,718 putative leptocylindracean V4 sequences belonged to Leptocylindraceae (Table 1). The query in GenBank with reference leptocylindracean sequences returned 46 V4 and six V9 sequences.

**Table 1:** Number of reads and OTUs obtained by clustering the BioMarKs sequences at 97% similarity cut-off.

Sequences	Nr of sequences		Nr of OTUs	
	V4	V9	V4	V9
All BioMarKs	1,476,249	195,944,951		
Diatoms	202,834 (13.7%^a^)	14,197,290 (7.2%^a^)		
Putative Leptocylindraceae (before removal)	51,378	1,526,145	51	844
Putative Leptocylindraceae (after removal of single- and doubletons)	50,718	1,525,527	12	403
Leptocylindraceae (after removal of single- and doubletons and false positives)	50,718 (25.0%^b^)	466,070 (3.3%^b^)	12	157
*L. aporus*	48,881	135,370	4	51
*L. convexus*	18	9,919	1	8
*L. danicus/hargravesii* c	1,394	271,960	3	68
Baffin Bay Clade^d^	4		1	
*L. minimus*	249	24,464	2	14
*T. belgicus*	172	24,357	1	16

a percentage over all BioMarKs sequences.

b percentage over all BioMarKs diatom sequences.

c ambiguously distinguishable in V4 sequences.

d identified only among V4 sequences.

### Taxonomic analysis

#### The V4 dataset

A total of 51 OTUs were identified among the 51,378 putative leptocylindracean V4 sequences from BioMarKs. Following singleton and doubleton removal, 50,718 sequences in 12 OTUs remained (Table 1). These sequences were aligned into 412 positions of which 144 were parsimony informative sites.

**Figure 1:**
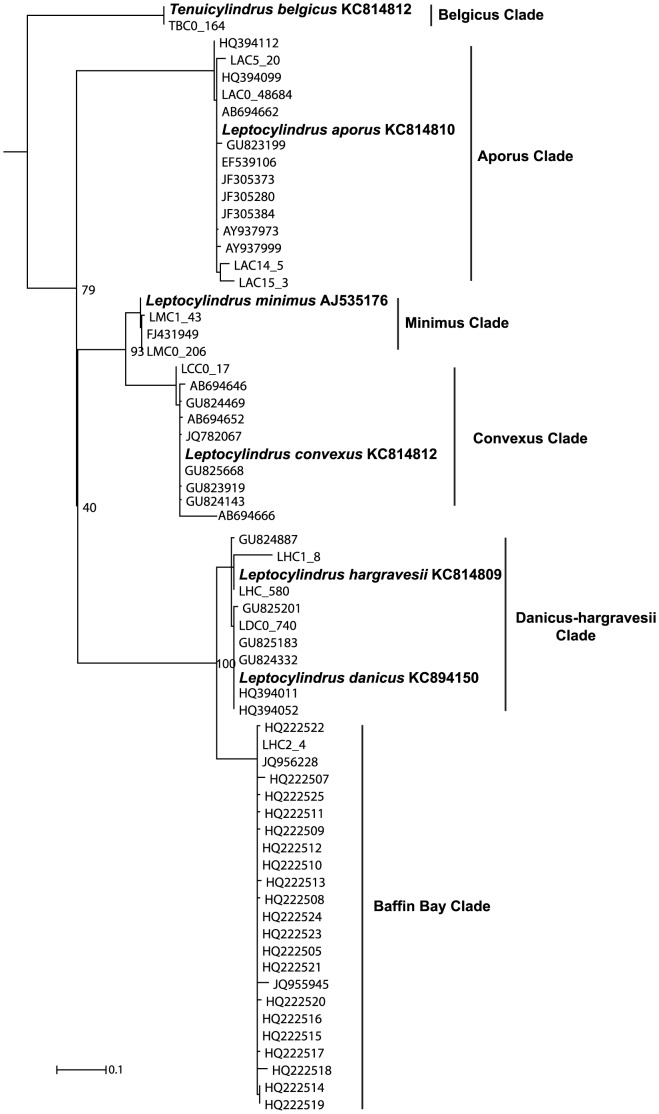
RAxML tree inferred from the alignment of 12 representative V4 sequences of leptocylindracean OTUs from the BioMarKs data, 46 leptocylindracean sequences from GenBank, and 134 reference sequences of bolidomonads, Leptocylindraceae and other diatoms, utilizing the GTRGAMMA base substitution model and Hill Climbing algorithm. *Bolidomonas pacifica* and *B. mediterranea* were designated as outgroups. All non-leptocylindracean reference sequences were pruned from the tree following tree construction (see Figure S1 for tree with outgroups included). Bootstrap values were inferred from 100 distinct alternative runs and values <50 are deleted. Each OTU is labelled as follows: the first letter denotes the first letter of the genus, the second letter, the first one of the species; the number denotes the cluster number (numbering starts from zero); the number after the underscore denotes the abundance of the OTU.

Within the ML phylogeny (Figure 1, all sequences of non-leptocylindracean taxa pruned away; Figure S2, outgroups included) all putative leptocylindracean sequences formed a clade as sister to a clade with all other diatoms, though without bootstrap support. Pennate diatoms formed a clade inside a grade of centric diatoms. In general, topology of the V4 tree resembled that of the SSU rDNA tree (Figure S3; constructed with complete or available information for the SSU rDNA region). Within the leptocylindraceans, six terminal clades were identified, i.e., clades that resolved internally into a polytomy. Five of these clades included reference sequences of known Leptocylindraceae species. Sequences in the clade including the reference sequence of *L. aporus* largely dominated the dataset (Table 1). References sequences of *L. danicus* and *L. hargravesii* were in the same clade, and the two taxa are considered together in the following, although several NGS V4 sequences were assigned to one or the other of the two species, given the higher uncertainty of these results. The sixth clade, without a reference sequence, included a single BioMarKs' OTU (with four sequences) and 22 GenBank sequences from Baffin Bay (Canada) [34]. Sequences in this clade, herewith defined as the ‘Baffin Bay Clade’, differed from those of *L. danicus/hargravesii* in 29 substitutions and seven insertion-deletions. The other 24 GenBank sequences belonged to clades including the reference sequences of *L. aporus* (10), *L. convexus* (7), *L. danicus/hargravesii* (6) and *L. minimus* (1), respectively.

#### The V9 dataset

A total of 844 OTUs were identified among the 1,526,145 putatively leptocylindracean V9 sequences from BioMarKs. Following removal of singletons and doubletons 1,525,527 sequences in 403 OTUs remained (Table 1). Alignment of these 403 representative sequences with six sequences from GenBank and the 97 reference sequences of bolidomonads, Leptocylindraceae and other diatoms resulted in a dataset of 224 positions. An initial ML-phylogeny resulted in non-monophyly for Leptocylindraceae as well as non-monophyly for the genus *Leptocylindrus*. Following removal of the false positives, the V9 alignment contained 165 OTUs and 188 positions, of which 76 parsimony-informative.

**Figure 2:**
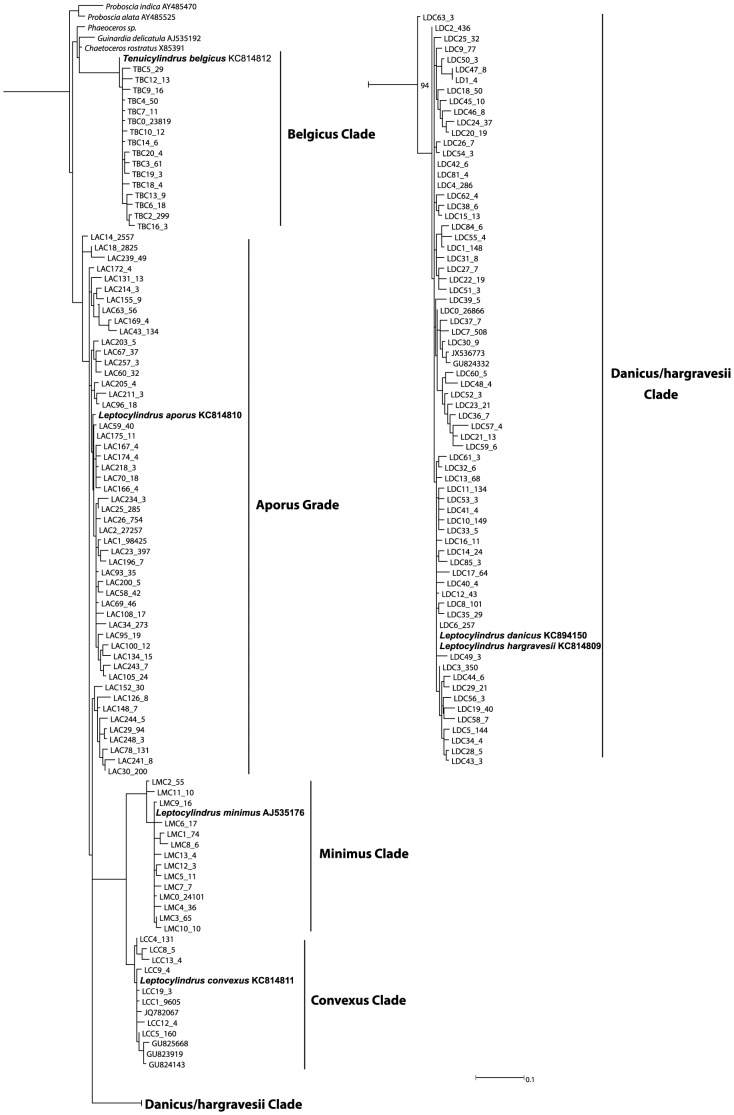
RAxML tree inferred from the alignment of 165 representative V9 sequences of leptocylindracean OTUs from the BioMarKs data, six leptocylindracean sequences from GenBank, and 96 reference sequences of bolidomonads, leptocylindraceans and other diatoms, utilizing the GTRGAMMA base substitution model and Hill Climbing algorithm. *Bolidomonas pacifica* and *B. mediterranea* were designated as outgroups. All non-leptocylindracean sequences were pruned from the tree following tree construction (see Figure S2 for tree with outgroups included). Bootstrap values were inferred from 100 distinct alternative runs and values <50 are deleted. OTU labels follow same principle as in Figure 1.

The ML V9 phylogeny was inferred from the 165 OTUs, six leptocylindracean sequences from GenBank, and 97 reference sequences of bolidomonads, leptocylindraceans and other diatoms (Figure 2, all sequences of non-leptocylindracean taxa pruned away; Figure S4, outgroups included). In this phylogeny, the branching pattern lacked any bootstrap support and Leptocylindraceae were not monophyletic, because the clade including *T. belgicus* was not sister to *Leptocylindrus*, thus deviating from the phylogeny shown from both the SSU rDNA (Figure S3) and V4 trees (Figure S2). Among the outgroups, neither pennates nor centrics were monophyletic (Figure S3). Nonetheless, five clades could be delineated for Leptocylindraceae, one of which included the V9 reference sequence of *L. danicus* and *L. hargravesii* (indistinguishable in this region) and the remaining four included each one reference sequence of the other known species in the family. Sequences in the clades including the reference sequences of *L. danicus-hargravesii* and *L. aporus* dominated the dataset (Table 1).

GenBank returned six V9 sequences, four of which belonged to the clade including the reference sequence of *L. convexus* and two to that of *L. danicus/hargravesii* (Table S2 in Tables S1). We were unable to identify a clade corresponding to the V4 Baffin Bay Clade because the GenBank sequences from this locality did not include the V9 region.

### Sequence distribution in the samples

For the distribution and geographic allocation of sequences, we assigned a sequence to a particular species if it grouped within the clade in which also the reference sequence of that particular species was recovered. In this exercise, *L. danicus* and *L. hargravesii* were treated as a single species because their V9 sequences do not differ.

Within the plankton and sediment samples, leptocylindracean V4 sequences were taxonomically more diverse and often more abundant in the cDNA than in the DNA, especially in samples containing high numbers of those sequences (Figure S5 A–C, Table S6–7 in Tables S1; at <0.05 significance). In the vast majority of plankton samples, the size fraction 3–20 µm showed the highest relative proportion of leptocylindracean V4 sequences as well as the best taxonomic coverage (Figure S6 A–C, Table S3 in Tables S1). Surface plankton samples usually contained a higher proportion of leptocylindracean V4 sequences as well as a higher taxonomic diversity than plankton samples taken at depth (Figure S6 A–C, Table S3 in Tables S1).

The V9 dataset, which was mainly obtained from cDNA templates from surface samples and cDNA and DNA templates from sediments, showed variable abundance in the three plankton size-fractions (Figure S8 A–C, Table S4 in Tables S1), except for sequences attributed to *L. minimus* and *T. belgicus*, which were abundant in the 0.8–3 µm fraction (Figure S8 E & F, Table S4 in Tables S1).

In both the V4 and the V9 datasets, species detected in sediment samples were in general the same as those recovered in the plankton at the same site, but not all species found in the plankton were retrieved in the sediments at the same site.

### Geographic allocation of sequences

**Figure 3:**
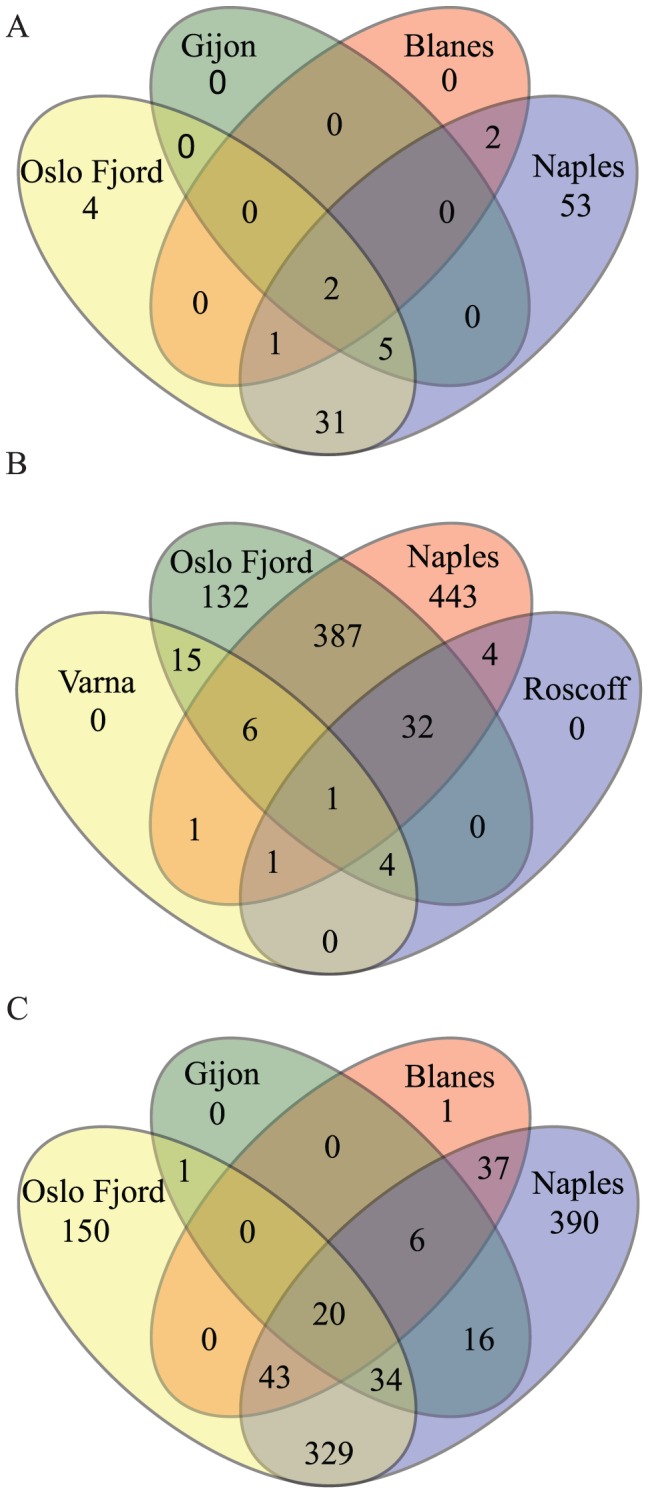
Venn diagrams showing the number of site-specific and shared OTUs among the six sampling stations. (A) V4 at Naples, Oslo Fjord, Gijon and Blanes (B) V9 at Varna, Oslo Fjord, Naples and Roscoff (C) V9 at Oslo Fjord, Gijon, Blanes and Naples. Venn diagrams for V9 have been split into two figures to compare OTU distribution among the sequence-abundant stations Naples and Oslo and the other four stations.

Clustering of MSA sequences (all size fractions and two depths, cDNA and DNA) into OTUs at 97% showed 187 and 1307 OTUs for the V4 and V9 datasets, respectively (Figure 3 A–C). Upon removal of singletons and doubletons 150 and 1027 OTUs were retained. Venn diagrams for the V4 dataset showed that the Oslo Fjord and Naples samples shared the highest numbers of OTUs, and also exhibited many site-specific OTUs (Figure 3A). The samples from Gijon and Blanes contained 7 and 5 OTUs only, which were shared with the samples from Oslo Fjord and Naples (Figure 3A). Venn diagrams for the V9 data showed that, again, Oslo Fjord and Naples exhibited the highest numbers of OTUs, shared the maximum number of OTUs and contained many site-specific OTUs (Figure 3B). Of the 28 OTUs found at Varna, 15 were shared only with Oslo Fjord and 12 with both Naples and Roscoff (Figure 3C).

**Figure 4:**
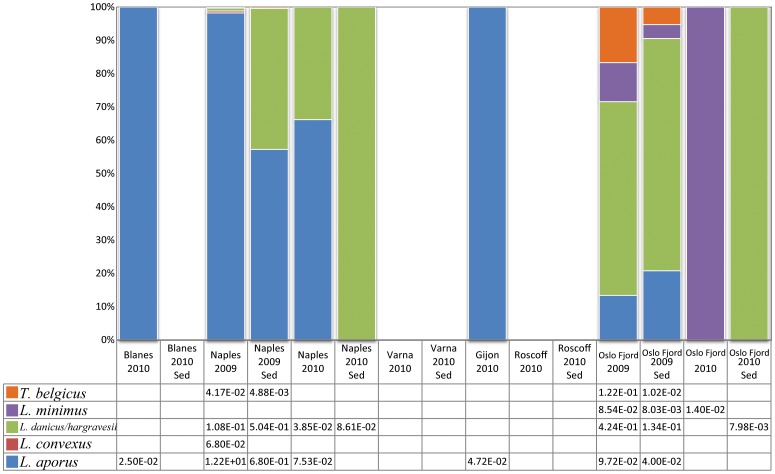
Relative abundance of NGS V4 Leptocylindraceae sequences at the six stations. Data for plankton samples were inferred from the surface cDNA results normalised over the total number of sequences obtained for the sample and the average of the three size fractions (mean relative frequency). Data for sediment samples were inferred from cDNA template based sequences (relative frequency).

V4 sequences belonging to leptocylindracean taxa were observed in the plankton samples of four of the six sites, and in benthos samples of two of the five sites (Figure 4, Table S3 in Tables S1, all size fractions, cDNA and DNA). Sequences of individual species at each station were generally uncommon or rare, constituting <0.5% of the total BioMarKs sequences (also including metazoans), except for *L. aporus*, which was abundant in the 2009 plankton sample of Naples. This species provided the bulk of the leptocylindracean sequences, constituting in all ca 25% of the diatom sequences in the overall V4 dataset (Table 1).

Among the stations, the autumn 2009 plankton samples obtained from Naples and Oslo Fjord and the autumn 2009 sediment sample from Oslo Fjord were the most diverse, with V4 sequences close or identical to reference sequences of four species. This result may depend on the fact that those samples were sequenced multiple times, and a higher number of sequences probably resulted in a better coverage of the diversity at those sites. Based on the V4 dataset, sequences attributed to *L. aporus* were present at many stations, while those of *L. convexus* were found only in the 2009 plankton sample of Naples. *Leptocylindrus minimus* V4 sequences were also rare and found only in Oslo Fjord samples. Sequences belonging to the Baffin Bay Clade were encountered only in the sediment samples of Oslo Fjord, and even there they were exceedingly rare (two in the 2009 sample, and two in the 2010 sample).

**Figure 5:**
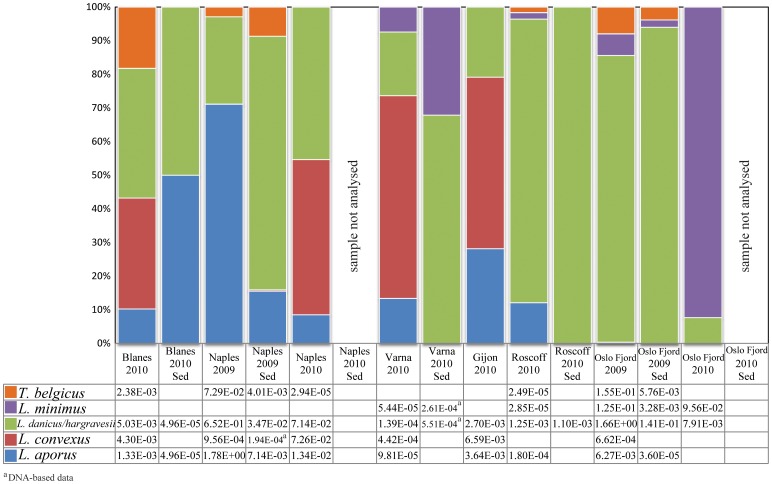
Relative abundance of NGS V9 Leptocylindraceae sequences at the six stations. Data for plankton samples were inferred from the surface cDNA results normalised over the total number of sequences obtained for the sample and the average of the three size fractions (mean relative frequency). Data for sediment samples were inferred from cDNA template based sequences (relative frequency), with the exception of sediment samples from Naples 2009 and Varna 2010.

With higher sequencing depth, V9 sequences attributable to Leptocylindraceae were present at all six stations, notably also in the plankton and sediment samples from Varna (May 2010) and Roscoff (April 2010) as well as in the sediment sample of Blanes (Figure 5, Table S4 in Tables S1), where the V4 dataset did not show any leptocylindracean sequences. *Leptocylindrus convexus* was conspicuous among the V9 sequences from plankton samples gathered at Blanes, Naples, Gijon and Varna (all in 2010), but basically absent from the V4 sequences obtained from the same samples or any other sample (Figure 4 & 5). The same applies to *Tenuicylindrus belgicus*, which was relatively abundant in the V9 samples from Blanes plankton samples but was not detected in the V4 dataset at that site (Table S5 in Tables S1; at <0.05 significance). The far larger number of leptocylindracean sequences, or diatom sequences in general, in the V9- versus the V4- dataset could not account for this difference in all cases (Table S5 in Tables S1; at <0.05 significance).

Like for the V4 dataset, V9 sequences of individual species in each site were rare (<0.2% of total sequences in respective samples), with the exception of a few samples from Naples and Oslo Fjord, where they attained more than 1.5%, providing most of the fraction of leptocylindracean sequences (3.3%) in the total diatom V9 dataset (Table 1). Among the stations, the autumn 2009 plankton sample of Oslo Fjord was the most diverse, with representatives of all the known species, while Blanes, Naples, Roscoff and Varna samples were equally diverse, with V9 sequences of four of the five species. V9 sequences attributed to *L. aporus* and *L. danicus/hargravesii* were found at all sites, while sequences of the latter species were found in all samples and dominated in several of them. V9 sequences of *L. minimus* and *T. belgicus* were less widely distributed, being found only at four sites (Figure 5).

Sequences belonging to clades including *L. aporus*, *L. convexus* and *T. belgicus* reference sequences were detected in plankton samples obtained when seawater temperatures ranged between 12.5 and 22.8°C (Table S1 in Tables S1). However, they were absent from the June 2010 water column samples from Oslo Fjord, where the temperature was within this range as well. Sequences attributable to *T. belgicus* were present in the autumn 2009 samples from Naples (22.8°C) and from Oslo Fjord (15.5°C).

**Figure 6:**
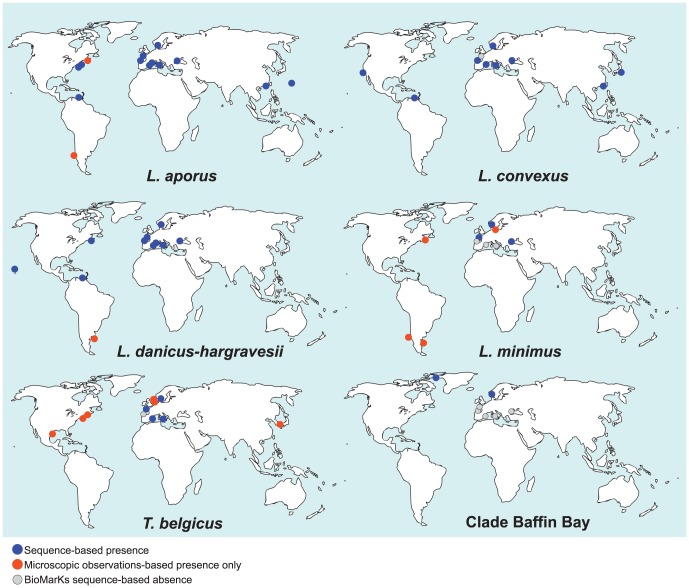
Distribution maps of Leptocylindraceae species inferred from NGS V4 and V9 sequences in the BioMarKs and GenBank datasets (blue dots), plus reliable microscopy images (red dots). Absence of finding in the BioMarKs dataset is represented by grey dots. Records for the microscopic observation reports of species presence are provided in supplementary material.

A Blast search in the GenBank database for leptocylindracean sequences retrieved a restricted number of sequences and added only a few geographic locations at which these sequences were recorded (Table S4 in Tables S1). Sequences of *L. aporus* were detected in the North Atlantic Ocean, the Western Pacific Ocean and in Sagami Bay, Japan; those belonging to *L. convexus* were also detected in Sagami Bay, as well as in Monterey Bay and in the Caribbean Sea; those of *L. danicus* were found in the Caribbean Sea and near Honolulu, Hawaii, and those of *L. hargravesii* were observed in the Caribbean Sea. The distribution of leptocylindracean species inferred from GenBank sequences and BioMarKs V4 and V9 sequences is presented in Figure 6.

## Discussion

In this study we explored the diversity and distribution of individual species of a recently re-investigated diatom family, the Leptocylindraceae, by tracing their characteristic SSU rDNA sequences in environmental DNA datasets. A high number of NGS reads ascribed to Leptocylindraceae were recovered at six coastal European sites sampled during the BioMarKs project, and several sequences were also retrieved from other locations around the world, using geo-localized sequence data from GenBank. Most leptocylindracean sequences were identical or similar to the reference sequences of the six described species, corroborating the species diversity assessment performed with culture-based methods in the Gulf of Naples (GoN) [7]. At least one novel group of sequences was identified, which probably belongs to an undescribed *Leptocylindrus* species. Regarding the biogeography of the Leptocylindraceae, this overview shows that all species described from the GoN are distributed across European seas. In the following sections, we discuss our findings highlighting the value of the DNA metabarcoding approach to assess diatom species diversity and distribution while pointing at some constraints in terms of the markers selected, the number of sequences obtained and the sampling strategy.

### Diversity assessment

Studies based on environmental DNA usually uncover a much higher biodiversity than those based on observations on fixed samples and even those on strain cultivation and genetic and morphological characterization. Molecular approaches of cultured taxa already revealed considerable cryptic and pseudo-cryptic diversity in several diatom species investigated so far [35]–[37]. Results of environmental DNA amplification and NGS usually show an even higher level of diversity, which however poses some interpretation problems.

Most of the BioMarKs leptocylindracean V4 sequences generated with the 454 technology were identical or highly similar to the leptocylindracean reference sequences. These V4 sequences grouped into a series of clades, all of which - except the Baffin Bay Clade - included one or two – in the case of *L. danicus*/*hargravesii* - reference sequences of known species. Each of these clades exhibited no further internal phylogenetic or geographic structure and the number of OTUs recovered within each of these clades was low (1 to 4). Notably, the reference sequences of the closely related species *L. danicus* and *L. hargravesii* were resolved in different OTUs, supporting the notion that these two OTUs represent different species. Basically, the leptocylindracean diversity in the European-wide BioMarKs V4 dataset corroborates the conclusion in Nanjappa et al. [7] inferred from the sequence diversity among Neapolitan strains that leptocylindracean species diversity is low. The GenBank V4 sequences from sites in and outside Europe further support this conclusion because all were identical to or a few base pairs different from those already revealed among the BioMarKs sequences. Additionally, the GenBank sequences provided further support to the Baffin Bay Clade, which likely represents an undescribed species.

In the BioMarKs V9 sequences, the 97% cut-off showed massive micro-variation and a far higher number of OTUs than the sequences in the V4 dataset. Moreover, bootstrap support for clades with these OTUs was generally poor or non-existent and neither did they show any geographic structure. Nevertheless, the reference sequences were resolved close to an OTU or a set of OTUs containing high numbers of sequences with an unresolved sprawl of OTUs containing low numbers of sequences.

Extensive micro-variability seems to be a characteristic of NGS data, showing up even within individual species [38]. As a matter of fact, the Sanger SSU rDNA sequencing of clonal cultures from the GoN belonging to each of the known species showed little or no internal variation in the V4 and V9 region [7]. Yet the documented OTU diversity, especially in the V9 dataset, could reflect a) rare intra-individual or intra-population sequence variation, and/or b) species that are exceedingly rare, inconspicuous or difficult to maintain in culture and are, therefore, unlikely to be detected using cell isolation and cultivation methods, and/or c) sequencing errors. The higher micro-variation in the V9 dataset could in theory result from the fact that most V9 sequences were generated from cDNA, which requires an extra processing step, namely reverse transcription of rRNA into cDNA. The existence of the closely related pseudo-cryptic species *L. danicus* and *L. hargravesii* demonstrates that groups of closely related species do exist in Leptocylindraceae and may escape detection, even using the V4 marker. Therefore, the use of more variable markers in DNA metabarcoding is needed to check our inference that the diversity in the family Leptocylindraceae is low.

To the aims of this paper, in further course of the discussion each clade as delineated in Figure 1 and 2 is considered as a species, and if it contains reference sequences of one or more species, then it belongs to those particular species. We assume that a sequence from a site represents a particular species at that site if this sequence groups within the largest possible clade in which also the reference sequence of the species is recovered. However, *L. danicus* and *L. hargravesii* are treated as a single species because they have very similar V4 and identical V9 sequences.

### Detection of Leptocylindraceae in the BioMarKs datasets

At the outset, the whole BioMarKs Illumina V9 dataset for protists was 130 times larger than the corresponding 454 V4 dataset, but the ratio decreased to ca. 92 times when only leptocylindracean sequences were taken into account. This difference could be due to a minor bias of the V9 primers against amplifying leptocylindracean sequences. Neither the V4 nor the V9 region of Leptocylindraceae is markedly longer than those of other stramenopiles, and no inserts have been detected [7], rendering amplification bias improbable. A possible explanation for the observed difference could be exclusion of a high number of leptocylindracean V9 sequences (false negatives) due to the relatively low phylogenetic resolution offered by the shorter V9 region.

Despite the lower proportion of leptocylindracean sequences in the V9 as compared to the V4 protist datasets, the Illumina V9 dataset was still much larger than the 454 V4 dataset, allowing the detection of known leptocylindracean species in more plankton and sediment samples from the various stations. In fact assignable V9 sequences were present in samples in which not even a single sequence attributable to the family could be detected in the V4 dataset. In addition, in some samples the high numbers of V9 sequences attributed to species missing in the V4 dataset indicate that those species were abundant enough to be detected also at the sequencing depth of the 454 approach (results drawn within the purview of single replicate but with statistical support). Bias of the V4 primers against amplifying the V4 region in certain leptocylindracean species does not explain the absence of sequences because, at least in silico, all the primers fitted perfectly the target sites in the reference SSU rDNA sequences of all the leptocylindracean species, including those of *L. convexus*, which was virtually absent from the V4 dataset.

The advantages of the V9 region in terms of species detection should however be weighed against the lower resolution allowed by such a short SSU rDNA region, which for example, cannot distinguish between closely related *L. danicus* and *L. hargravesii*. This lower resolution can be an issue in recently diversified diatom genera. Finally, the lower performance of V9 versus V4 in producing reliable distance trees can also constitute a hindrance to the detection of new taxa for which no reference sequences are available yet.

In the V4 dataset, which includes both the rDNA and cDNA templates, leptocylindracean sequences are generally more prominent among those generated from cDNA than from rDNA, suggesting that cells were physiologically active during sampling. Peculiarly, the predominance of cDNA sequences was observed also in some sediment samples. If leptocylindraceans were present there mainly as resting cells, then one would instead expect a lower proportion of ribosomal RNA (or cDNA generated from it) versus rDNA. Probably, the higher proportion of leptocylindracean cDNA sequences also those sediment samples might result from vegetative cells that just settled out of the water column. Alternatively, leptocylindracean cells could merely possess low numbers of rDNA copies in their genomes. rDNA copy numbers differ considerably among species, rendering estimations of the numbers of individuals per species from NGS data challenging [38], [39].

Size fractionation in NGS studies has the objective to separate organisms based on their dimensions and explore sequence diversity in each of the fractions separately. However, in the case of Leptocylindraceae, sequences were recovered in each of the size fractions (0.8–3 µm, 3–20 µm and 20–2000 µm) obtained in BioMarKs. The valve diameter in Leptocylindraceae (2–12 µm) explains their generally higher abundance in the intermediate size-fraction, while cells often longer than 20 µm and chains attaining even several hundred µm length [7] account for those organisms trapped when settling in girdle-view onto the 20 µm filter surface. In addition, when 20 µm-filters get clogged, the filtrate itself also traps small cells. On the other hand, the smallest cells may pass, with the valve head-on, through the 3 µm-pore, and so can the content of ruptured cells. The presence of sequences belonging to individual species in different size fractions is hence hardly predictable, as it depends on shape and size of cells and chains and on the propensity of the latter to fall apart, but also on the density of the plankton community, on the abundance of certain cell sizes therein and on the net-tow speed. A similarly wide distribution of sequences over all size fractions is expected for many other planktonic diatoms, due to their often elongated shapes and colonial habits as well as their variations in cell size resulting from clonal growth and sexual reproduction. Therefore, size fractionation in environmental NGS approaches may be of limited use if one wishes to focus on diatoms.

### Geographical distribution

Members of the centric diatom family Leptocylindraceae are recognizable in light microscopy. Yet, individual species therein have not been well defined until recently [7], and therefore, information about their distribution patterns is missing or unreliable. In several cases electron microscopy and even cultivation and strain sequencing are required to confirm species identity in the pseudo-cryptic species of the family. In this study, the identification of OTUs containing reference sequences of *Leptocylindrus* and *Tenuicylindrus* species in environmental DNA sequence datasets provided new information on the distribution of these species at several European and extra-European locations. However, the different markers and the different sequencing methods do have their shortcomings as has been illustrated above.

Based on our BioMarKs results, most species described in Nanjappa *et al.* [7] are widely distributed in European Seas, and the exploration of GenBank data has demonstrated their occurrence also in places outside Europe. The most widespread seems to be *L. aporus*, which was found at all BioMarKs sites as well as - according to GenBank data - along the French Mediterranean coast, in the North Pacific and on the North West Atlantic coast. It was actually in Narragansett Bay (North West Atlantic coasts) that the species was first described (as *L. danicus* var. *apora*) [7], [40]. *Leptocylindrus danicus* and/or *L. hargravesii* are also present at all BioMarKs sites, although distribution patterns of these two individual species cannot be explored in the V9 dataset. Based on V4 GenBank sequences, *L. danicus* might be more widespread, as it was detected along the French Mediterranean coast, the East Pacific and the Gulf of Mexico, while sequences of *L. hargravesii* were only found at the latter location. *Leptocylindrus hargravesii* is also much rarer in the GoN than *L. danicus* [7]. Yet both species were also first observed along the North-western Atlantic coast [7], [19], while GenBank sequences from that area, also obtained from environmental DNA, only revealed the presence of *L. aporus*. Indeed, lack of observation cannot be translated in absence for plankton microbes, and this may be true for NGS data as well, despite the sequence depth allowed by this approach. *Leptocylindrus convexus* also seems to be widespread based on GenBank sequences, although it was not retrieved at the Roscoff sampling site. As this species can also be identified in the light microscope based on the typically convex valve shape, data on its geographic range will probably accumulate also independently from metabarcoding in future years.

The large distribution ranges of the four above-mentioned species agree with the concept that microorganisms, including prokaryotes, unicellular eukaryotes, and small multicellular organisms, are cosmopolitan, which forms the basis for the hypothesis that “everything is everywhere, but the environment selects” [41], [42]. However recent studies have demonstrated that microorganisms may exhibit biogeographic patterns, although the rates of the underlying processes vary more widely than for macroorganisms [43]–[45]. Indeed the remaining two known species to be discussed, *L. minimus* and *Tenuicylindrus belgicus* were virtually absent from the GenBank dataset (only one sequence of the former from Roscoff) and also missing at several of the BioMarKs sites. *Tenuicylindrus belgicus* was absent from the Gijon (North Atlantic) and Varna (Black Sea) stations, whereas *L. minimus* was only found at the Oslo Fjord, Roscoff and Black Sea stations. In the case of *L. minimus*, a GenBank sequence and previous observations [7], [19], [46] show that it is also present along the North West US coast. *Leptocylindrus minimus* was the only species never found in the GoN in the previous survey of the genus [7], and it did not show up at any of the Mediterranean Sea sites in this first NGS survey either. Therefore, the species is probably restricted to colder waters at least in Europe. Its presence in the Black Sea could seem contradictory, yet in the latter area there are other examples of cold-water relict species, while quite unequivocal drawings confirm that it was also recognised in light microscopy [47]. Similarly, the possibly new species identified in this paper also appeared only in sequences from Baffin Bay (GenBank) and Oslo Fjord (BioMarKs). These results are in agreement with observations based on cultured strains of *Skeletonema* species, which also include both widespread representatives and species with a restricted distribution pattern [6]. The same pattern is also evident at intraspecific level, as many OTUs were shared e.g. between Oslo Fjord and Naples, while a fair number of them were specific to one site. Interestingly, a higher number of OTUs and a higher proportion site-specific versus shared OTUs were identified in the latter site as compared to the Oslo Fjord samples, suggesting a higher intraspecific diversity in the GoN.

### Seasonality

The apparently restricted geographic range found for *L. minimus*, *T. belgicus* and the Baffin Bay Clade could also be the result of a short season of occurrence for these species, which may have not been covered in the BioMarKs sampling plan, which included at most one or two sampling occasions for a few days per site. The sequencing depth of the NGS approach, especially with the Illumina technology, should overcome this problem and detect also species present at very low concentrations. However results obtained in Oslo Fjord and GoN on two different sampling dates only partially support this hypothesis. In the GoN, according to previous observations [7], *L. danicus* and *L. convexus* occur from late autumn through mid-summer; *L. hargravesii*, in winter; *L. aporus*, in summer and autumn; and *T. belgicus* in late summer and autumn, whereas *L. minimus* is not found at all. The BioMarKs V4- and V9-data collected in autumn in the GoN agree with these observations, as they show *L. aporus* dominating in the October 2009 sample and also reveal the presence of *L. danicus/hargravesii*, *L. convexus* and *T. belgicus* on that date. The May 2010 plankton samples was expected to contain only *L. danicus/hargravesii* and *L. convexus* but the V9 datasets also showed many sequences of *L. aporus* and of *T. belgicus*, although the latter in very low numbers. Therefore, the sequencing depth in this case seemed to compensate the marked seasonality observed for these species through microscopy and strain isolation. By contrast, in the case of Oslo Fjord, all the six known species and the new Baffin Bay taxon were detected in the autumn 2009, but only *L. minimus* and of *L. danicus/hargravesii* sequences were obtained in spring 2010. This latter case definitely points at the need to include samples from different periods of the year when addressing the biogeography of species that may have pronounced seasonal patterns.

Whether occasional plankton sampling can cover the entire diversity of an area may also depend on the overwintering strategy of the species of interest, which could either persist in the water column at low concentrations or form resting stages or spores settling onto the seafloor. In the case of spore-formers, sediment sampling could effectively detect even species not found in the water column on a certain date. In the case of Leptocylindraceae, at least *L. danicus*, *L. hargravesii* and *L. minimus* are known to form benthic resting stages [7], [48], [49]. The finding of a few sequences belonging to the Baffin Bay Clade in the sediment of Oslo Fjord would confirm that sediment samples may reveal more diversity than the corresponding plankton samples by detecting species blooming in the water column outside the season of the plankton sampling. On the other hand, rather than a simple seed bank, the sediments are quite dynamic in terms of diatom composition, being largely influenced by the rain of cells from upper layers [50]. This explains why both spore formers and non-spore formers may be detected in sediments, as it was the case of the GoN, where all the species found in the water column were also detected in the sediments. The alternative explanation is that the absence of evidence is not evidence of absence of benthic resting cells or spores in the life cycle of those species. In some cases, however, sediment samples did not include spore-former species found on the same or in other dates in plankton samples at that site. For instance, the spore-former *L. minimus* was absent from the sediment samples of Roscoff. Possibly, those spores were very rare in the sediments, or the DNA extraction methods were not efficient enough to extract DNA from resting stages, which have a very thick and silicified shell. Therefore even the analysis of DNA from the sediments may fail to catch all the species diversity at one site, at least with the current methods.

## Conclusions

The present NGS-based DNA-metabarcoding study provides an overview of the species diversity of Leptocylindraceae and offers a glimpse into the biogeographic distribution of these species in European coastal waters. Additional information from GenBank showed the presence of several of these species also at sites outside Europe. A 454-sequencing exercise of a V4 fragment revealed clearly defined clades of leptocylindracean species, low sequence diversity within these clades, and restricted distribution patterns of the individual species. Instead, more massive Illumina-sequencing of a V9 fragment revealed higher sequence variation, but the clades were less well-delineated. Yet, the V9 dataset permitted detection of species at sites where the V4 data failed to do so. Nonetheless, the number of new species detected using NGS data did not increase markedly, supporting the idea that the family Leptocylindraceae, which occupies a basal position in the phylogeny of the diatoms, is species poor. Information from studies in different parts of the word is needed to increase the resolution of geographic patterns and to search for additional diversity.

Understanding the diversity, biogeography and ecological role of protists depends on the degree of correlation between morphological and molecular characters. Morphological analysis involves extensive observation, measurement, comparison and documentation whereas estimating and interpreting species richness from the molecular data obtained through NGS of environmental samples depends on the choice made in term of molecular marker as well as on the cut-off values used for delineating OTUs. Among the variety of choices available for genetic markers selection to estimate the diversity of species in environmental samples, the widely used SSU rDNA, V4 and V9 regions provided similar taxon discrimination, but V4 produced more reliable distance trees, while V9 showed a higher detection power in the case of the target diatoms of this study.

Our study has shown that data obtained in NGS-based DNA-metabarcoding exercises can be mined to assess species diversity, even within ancient lineages, and to establish the biogeographic pattern of the delineated species. Continued efforts in the choice of adequate marker regions and the improvement of sampling strategies and bioinformatic tools used for analysing metabarcoding data are needed to improve the interpretation of species richness in environmental samples thereby fostering studies of biogeography and ecology of marine microbes.

## Supporting Information

Figure S1 **Rarefaction curves at different similarities inferred from Leptocylindraceae sequences from the BioMarKs dataset containing reads from pooled fractions.** (A) V4 (B) V9.
(EPS)

Figure S2 **RAxML phylogenetic tree showing the position of the V4 NGS leptocylindracean sequences retrieved from BioMarKs and GenBank in relation to V4 reference sequences of Bacillariophyta and Bolidophyceae.** *Bolidomonas pacifica* and *B. mediterranea* were selected as outgroups. Tree inference was derived from GTRGAMMA base substitution model and Hill Climbing algorithm. Bootstrap values were inferred from 100 distinct alternative runs and values of <50 are deleted. Each OTU is labelled as follows: the first letter denotes the first letter of the genus, the second letter, the first one of the species; the number denotes the cluster number (numbering starts from zero); the number after the underscore denotes the abundance of OTU.
(EPS)

Figure S3 **RAxML phylogenetic tree inferred from whole SSU rDNA reference sequences of Bacillariophyta and Bolidophyceae.** *Bolidomonas pacifica* and *B. mediterranea* were selected as outgroups. Tree inference was derived from GTRGAMMA base substitution model and Hill Climbing algorithm. Bootstrap values were inferred from 100 distinct alternative runs and values of <50 are deleted.
(EPS)

Figure S4 **RAxML phylogenetic tree showing the position of the V9 NGS leptocylindracean sequences retrieved from BioMarKs and GenBank in relation to V9 reference sequences of Bacillariophyta and Bolidophyceae.** *Bolidomonas pacifica* and *B. mediterranea* were selected as outgroups. Tree inference was derived from GTRGAMMA base substitution model and Hill Climbing algorithm. Bootstrap values were inferred from 100 distinct alternative runs and values of <50 are deleted. OTU-labels follow same principle as in Fig. S2.
(EPS)

Figure S5 **Comparative abundance of V4 NGS leptocylindracean sequences obtained from the DNA and cDNA based template, inferred from surface plankton sample and 3–20 µm size fraction.** (A) *L. aporus* (B) *L. danicus/hargravesii* (C) *T. belgicus*. Asterisks represent samples with <10 sequences in V4, shown for completeness although the proportions are not reliable.
(EPS)

Figure S6 **Comparative abundance of V4 NGS leptocylindracean sequences in the three size fraction inferred from surface plankton sample and cDNA template.** (A) *L. aporus* (B) *L. danicus/hargravesii* (C) *L. minimus* (D) *T. belgicus*. Asterisks represent samples with <10 sequences in V4, shown for completeness although the proportions are not reliable.
(EPS)

Figure S7 **Comparative abundance of V4 NGS leptocylindracean sequences in the water column inferred from plankton sample and cDNA template.** (A) *L. aporus* (B) *L. danicus/hargravesii* (C) *L. minimus* (D) *T. belgicus*.
(EPS)

Figure S8 **Comparative abundance of V9 NGS leptocylindracean sequences in the three size fraction inferred from surface plankton sample and cDNA template.** (A) *L. aporus* (B) *L. convexus* (C) *L. danicus/hargravesii* (D) *L. minimus* (E) *T. belgicus*. Asterisks represent samples with <100 in sequences V9, shown for completeness although the proportions are not reliable.
(EPS)

Tables S1 **This file includes Table S1–S7.**
(XLSX)

Methods S1 **Supplementary methods.**
(DOCX)
