# Supplementary material for: Assessment of Species Diversity and Distribution of an Ancient Diatom Lineage Using a DNA Metabarcoding Approach
Source: PLoS One. 2014 Aug 18;9(8):e103810. doi: 10.1371/journal.pone.0103810 (PMC4136930; doi:10.1371/journal.pone.0103810)
Supplement: Methods S1 — Supplementary methods. (DOCX) [file pone.0103810.s010.docx]

**Supplementary methods.** The rationale for setting the sequence identity cut-off value at the low value of 0.90 was a considerable overlap of the similarities between, on one hand, pairs of highly different leptocylindracean sequences, and on the other hand, sequences of leptocylindraceaen and non-leptocylindracean species. Pairwise dissimilarities among the V4 regions varied within the ranges of 0.0025–0.2066 among *Leptocylindrus* spp., 0.01749–0.2168 between *Leptocylindrus* spp. and *T. belgicus,* and 0.1505–0.2303 between in- and outgroups. Pairwise dissimilarities among the V9 regions varied within the ranges of 0–0.1408 among *Leptocylindrus* spp., 0.1619–0.2038 between *Leptocylindrus* spp. and *T. belgicus,* and 0.0846–0.2241 between in- and outgroups. The 90% similarity cut-off value allowed inclusion of many false positives (non-leptocylindracean sequences, at times closer to other diatom or bolidomonad sequences), but minimized the risk of excluding false negatives (dissimilar sequences belonging to yet unknown leptocylindracean species). Pairwise similarities for this comparison were computed using PAUP* version 4.0b10 [[25](#_ENREF_25)].

**Supplementary Figure legends**

Figure S1. Rarefaction curves at different similarities inferred from Leptocylindraceae sequences from the BioMarKs dataset containing reads from pooled fractions. (A) V4 (B) V9

Figure S2. RAxML phylogenetic tree showing the position of the V4 NGS leptocylindracean sequences retrieved from BioMarKs and GenBank in relation to V4 reference sequences of Bacillariophyta and Bolidophyceae. *Bolidomonas* *pacifica* and *B. mediterranea* were selected as outgroups. Tree inference was derived from GTRGAMMA base substitution model and Hill Climbing algorithm. Bootstrap values were inferred from 100 distinct alternative runs and values of <50 are deleted. Each OTU is labelled as follows: the first letter denotes the first letter of the genus, the second letter, the first one of the species; the number denotes the cluster number (numbering starts from zero); the number after the underscore denotes the abundance of OTU.

Figure S3. RAxML phylogenetic tree inferred from whole SSU rDNA reference sequences of Bacillariophyta and Bolidophyceae. *Bolidomonas* *pacifica* and *B. mediterranea* were selected as outgroups. Tree inference was derived from GTRGAMMA base substitution model and Hill Climbing algorithm. Bootstrap values were inferred from 100 distinct alternative runs and values of <50 are deleted.

Figure S4. RAxML phylogenetic tree showing the position of the V9 NGS leptocylindracean sequences retrieved from BioMarKs and GenBank in relation to V9 reference sequences of Bacillariophyta and Bolidophyceae. *Bolidomonas* *pacifica* and *B. mediterranea* were selected as outgroups. Tree inference was derived from GTRGAMMA base substitution model and Hill Climbing algorithm. Bootstrap values were inferred from 100 distinct alternative runs and values of <50 are deleted. OTU-labels follow same principle as in Fig. S2.

Figure S5. Comparative abundance of V4 NGS leptocylindracean sequences obtained from the DNA and cDNA based template, inferred from surface plankton sample and 3–20µm size fraction. (A) *L. aporus* (B) *L. danicus/hargravesii* (C) *T. belgicus.* Asterisks represent samples with <10 sequences in V4, shown for completeness although the proportions are not reliable.

Figure S6. Comparative abundance of V4 NGS leptocylindracean sequences in the three size fraction inferred from surface plankton sample and cDNA template. (A) *L. aporus* (B) *L. danicus/hargravesii* (C) *L. minimus* (D) *T. belgicus.* Asterisks represent samples with <10 sequences in V4, shown for completeness although the proportions are not reliable.

Figure S7. Comparative abundance of V4 NGS leptocylindracean sequences in the water column inferred from plankton sample and cDNA template. (A) *L. aporus* (B) *L. danicus/hargravesii* (C) *L. minimus* (D) *T. belgicus*

Figure S8. Comparative abundance of V9 NGS leptocylindracean sequences in the three size fraction inferred from surface plankton sample and cDNA template. (A) *L. aporus* (B) *L*. *convexus* (C) *L. danicus/hargravesii* (D) *L. minimus* (E) *T. belgicus.* Asterisks represent samples with <100 in sequences V9, shown for completeness although the proportions are not reliable.

Records of species presence in a geographic location

***L. aporus***

1. French III FW, Hargraves PE (1986) Population dynamics of the spore-forming diatom *Leptocylindrus danicus* in Narragansett Bay, Rhode Island. Journal of Phycology 22: 411-420.

2. Rivera P, Cruces F, Clement A (2002) *Leptocylindrus minimus* Gran (Bacillariophyceae): morphology and distribution in Chile. Gayana Botánica 59: 7-11.

***L. danicus-hargravesii***

1. Sunesen I, Sar EA (2007) Diatomeas marinas de aguas costeras de la provincia de Buenos Aires (Argentina). III Géneros potencialmente nocivos *Asterionellopsis, Cerataulina, Ceratoneis* y *Leptocylindrus*. Revista Chilena de Historia Natural 80: 493-507.

2. French III FW, Hargraves PE (1986) Population dynamics of the spore-forming diatom *Leptocylindrus danicus* in Narragansett Bay, Rhode Island. Journal of Phycology 22: 411-420.

***L. minimus***

1. Kraberg A, Baumann M, Dürselen CD (2010) Coastal phytoplankton: photo guide for Northern European seas. München: Verlag Dr. Friedich Pfeil. 204 p.

2. Hargraves PE (1990) Studies on marine plankton diatoms. V. Morphology and distribution on *Leptocylindrus minimus* Gran. Nova Hedwigia Beiheft 100: 47-60.

3. Sunesen I, Sar EA (2007) Diatomeas marinas de aguas costeras de la provincia de Buenos Aires (Argentina). III Géneros potencialmente nocivos *Asterionellopsis, Cerataulina, Ceratoneis* y *Leptocylindrus*. Revista Chilena de Historia Natural 80: 493-507.

4. Rivera P, Cruces F, Clement A (2002) *Leptocylindrus minimus* Gran (Bacillariophyceae): morphology and distribution in Chile. Gayana Botánica 59: 7-11.

***T. belgicus***

1. Kraberg A, Baumann M, Dürselen CD (2010) Coastal phytoplankton: photo guide for Northern European seas. München: Verlag Dr. Friedich Pfeil. 204 p.

2. Meunier A (1915) Microplankton de la mer flamande. Bruxelles: Memories du Mesee Royal D'Histoire Naturelle de Belgique. 47 p.

3. University of Yokohama. 2012. Microflora/fauna of Yokohama. Available at: <http://www.biol.tsukuba.ac.jp/~algae/YMFF/Bacillariophyceae/Leptocylindrus_minimus/index.html> (accessed November 13, 2012).

4. Plankton.net, Alfred Wegener Institute, Available at: <http://hdl.handle.net/10013/de.awi.planktonnet.image.12696> (accessed on January 30, 2014)

5. LUMCON's guide to phytoplankton from Louisiana estuarine and coastal waters. Available at: <http://phytoplanktonguide.lumcon.edu/default.asp> (accessed January 30, 2014)
